# Prospective study of a rotating gantry with scanning beams of carbon-ion radiotherapy for choroidal malignant melanoma

**DOI:** 10.1093/jrr/rraf030

**Published:** 2025-06-10

**Authors:** Masaru Wakatsuki, Hirokazu Makishima, Shuri Aoki, Nao Kobayashi, Hiroshi Tsuji, Hitoshi Ishikawa, Shigeru Yamada, Atsushi Mizota

**Affiliations:** QST Hospital, National Institutes for Quantum Science and Technology, 4-9-1 Anagawa, Inage-ku, Chiba 263-8555, Japan; QST Hospital, National Institutes for Quantum Science and Technology, 4-9-1 Anagawa, Inage-ku, Chiba 263-8555, Japan; QST Hospital, National Institutes for Quantum Science and Technology, 4-9-1 Anagawa, Inage-ku, Chiba 263-8555, Japan; Radiation Oncology Department, Cancer Institute Hospital, Japanese Foundation for Cancer Research, 3-8-31, Ariake, Koto-ku, Tokyo 135-8550, Japan; QST Hospital, National Institutes for Quantum Science and Technology, 4-9-1 Anagawa, Inage-ku, Chiba 263-8555, Japan; QST Hospital, National Institutes for Quantum Science and Technology, 4-9-1 Anagawa, Inage-ku, Chiba 263-8555, Japan; QST Hospital, National Institutes for Quantum Science and Technology, 4-9-1 Anagawa, Inage-ku, Chiba 263-8555, Japan; QST Hospital, National Institutes for Quantum Science and Technology, 4-9-1 Anagawa, Inage-ku, Chiba 263-8555, Japan; Department of Ophthalmology, Teikyo University School of Medicine, 2-11-1 Kaga, Itabashi-Ku, Tokyo 173-8606, Japan

**Keywords:** choroidal malignant melanoma, carbon-ion radiotherapy, rotating gantry, scanning beams, particle therapy

## Abstract

To evaluate the safety of a rotating gantry with scanning beams of carbon-ion radiotherapy (C-ion RT) for choroidal malignant melanoma. A prospective study of C-ion RT using a rotating gantry with scanning beams for choroidal malignant melanoma was initiated at the National Institute for Quantum Science and Technology, QST Hospital in March 2018. The inclusion criteria were as follows: (i) clinically diagnosed ocular/choroidal malignant melanoma; (ii) tumor measurable by imaging tests; (iii) score of 0–2 on the Eastern Cooperative Oncology Group Performance Status scale and (iv) ability to provide consent for treatment. All patients received 68 Gy in four fractions of C-ion RT by a rotating gantry with scanning beams. Between April 2018 and July 2019, 21 patients were enrolled and underwent C-ion RT as planned. All 21 patients completed the treatment schedule and the 3-year follow-up period. The median duration of follow-up was 43 months (range, 35.2–54.6 months). Regarding late normal tissue responses, three of the 21 patients developed grade 2 neovascular glaucoma; however, no other late grade ≥2 acute toxicities were observed. During the 3-year study period, all 21 patients survived with no local recurrence; none of the patients underwent enucleation. Three cases showed liver metastasis. The 3-year local control, overall survival and eye-retention rates were all 100%. The results of this prospective study confirmed that the effectiveness and safety of this method are equivalent to those of conventional passive irradiation methods, although the number of cases was small. The results of this prospective study confirmed that the effectiveness and safety of this method are equivalent to those of conventional passive irradiation methods, although the number of cases was small.

## INTRODUCTION

Choroidal melanomas are the most frequent primary intraocular malignant neoplasms in adults, although it occurs less commonly than cutaneous melanomas or other cancers [1]. Traditionally, choroidal melanomas have been treated with enucleation of the eye. However, recent eye-conserving treatment approaches, such as local resection, photodynamic therapy, brachytherapy, stereotactic radiotherapy and particle radiotherapy have become the treatments of choice in many cases [2, 3]. Taking advantage of its high dose concentration, particle therapy has been indicated for large-sized choroidal melanoma and has shown good results. Proton therapy has been administered to treat choroidal malignant melanomas at many institutions, mainly in Europe and the United States, yielding satisfactory results [4–6]. Contrastingly, carbon-ion radiotherapy (C-ion RT) can offer better dose conformity and biological effects than proton therapy, although the difference in conformity depends on the depth of the target lesion [7]. In 1994, C-ion RT was initiated at the National Institute for Quantum Science and Technology (QST; formerly the National Institute of Radiological Sciences [NIRS]) in Japan. Carbon-ion beams have improved dose-localization properties, which produce significant effects on tumors while minimizing normal tissue damage. Moreover, they possess biological advantages due to their high relative biological effectiveness (RBE) at the Bragg Peak. The usefulness of C-ion RT for many diseases has been established, and the national health insurance coverage for many diseases is expanding in Japan [8–11]. A phase I/II dose-escalation study of C-ion RT for locally advanced or unfavorably located choroidal melanoma was initiated in 2001, and its long- term results have been reported [12, 13]. The results showed a local control rate of 93% and an eye retention rate (ERR) of 93% at 5 years, comparable to those of proton therapy. However, the incidence of grade 2 neovascular glaucoma (NVG) at 3 years was 41.6% and 13.9% for 1- and 2-portal irradiation, respectively. Reducing the incidence of glaucoma was deemed necessary to improve the rate of eye preservation and patient quality of life. Subsequently, in 2015, QST installed a rotating gantry with scanning beams, exploring superconducting magnet technology [14–17]. This gantry enables the irradiation of patients from multiple beam angles, providing greater flexibility. Previous studies comparing the dose distribution of scanning irradiation and conventional passive irradiation have shown that it is possible to reduce the skin dose for targets that are relatively close to the skin [18]. This suggests that it may also be useful in the treatment of choroidal malignant melanoma.

Thus, we conducted a prospective trial for choroidal malignant melanoma to evaluate the safety of the rotating gantry with scanning beams of C-ion RT. To the best of our knowledge, this is the first study using this technique for choroidal malignant melanoma. Furthermore, the trial aimed to confirm the safety of treatment methods different from conventional passive irradiation methods and to establish treatments with a reduced patient burden.

## MATERIALS AND METHODS

### Protocol and aims

A prospective study of C-ion RT using a rotating gantry with scanning beam for choroidal malignant melanoma was initiated at our institute in March 2018. This prospective study aimed to evaluate the safety and efficacy of C-ion RT using this new technique.

### Patient eligibility

The inclusion criteria for the patients and tumors were as follows: (i) clinically diagnosed ocular/choroidal malignant melanoma; (ii) tumor measurable by imaging tests; (iii) score of 0–2 on the Eastern Cooperative Oncology Group (ECOG) Performance Status scale and (iv) ability to provide consent for treatment. In case of minors, informed consent was obtained from their guardians.

The exclusion criteria were as follows: (i) evidence of extraocular lesions; (ii) presumed life expectancy of <6 months; (iii) active treatment-resistant infection within the irradiation field; (iv) active coexisting malignancy; (v) severe coexisting disease and (vi) clinically unfit to complete the study. Clinical evaluation included computed tomography (CT), magnetic resonance imaging (MRI), ultrasonography (US) and ophthalmoscopy for the diagnosis of the primary tumor as well as whole-body CT or ^18^F-fluorodeoxyglucose positron emission tomography (FDG-PET)/CT and liver MRI to rule out distant metastasis.

### Ethics

Approval for the present study was obtained from the Institutional Review Board (ID: 17–106). This study was registered with the University Hospital Medical Information Network Clinical Trials Registry (http://www.umin.ac.jp/ctr/index-j.htm; identification number: UMIN000031715).

### Endpoints and statistical analyses

The primary endpoint of this study was acute normal tissue toxicity, whereas the secondary endpoints were local control (LC), overall survival (OS), eye retention (ER) and late normal tissue toxicity.

Patients were followed up at 3-month intervals during the first 6 months after C-ion RT and then at 6-month intervals until 3 years after C-ion RT. MRI and CT of the orbit, US or MRI of the liver and CT of the lungs or FDG-PET/CT were performed every 6 months. Visual acuity (VA), intraocular pressure and visual field were evaluated during each ophthalmological examination. Acute normal tissue responses were scored using the Common Terminology Criteria for Adverse Events (version 4.0; 2009) for the first 3 months after C-ion RT. Late normal tissue responses were scored using the Radiation Therapy Oncology Group/European Organization for Research and Treatment of Cancer scoring system. No additional treatment after carbon-ion RT was allowed until recurrence was observed, and there were no regulations regarding treatment at the time of recurrence.

Local recurrence was defined as a substantial increase in the size of the tumor (in-field recurrence) or the appearance of a newly developed tumor adjacent to the primary lesion (marginal recurrence) as demonstrated using any imaging modality or ophthalmoscopy. LC, OS and ER curves were plotted using the Kaplan–Meier method.

### C-ion RT

The details of the treatment procedure have been previously reported [13, 19]. The patients were immobilized with a low-temperature thermoplastic restraining device (Shellfitter; Kuraray Co., Osaka, Japan) in the supine position. Their eyes were fixed using a gazing method developed at QST. A set of 1-mm-thick CT images was used for treatment planning with immobilization devices and a gazing light. CT-based treatment planning was performed for each patient using Xio-N2 (National Institute of Radiological Sciences, Chiba, Japan).

**Fig. 1:**
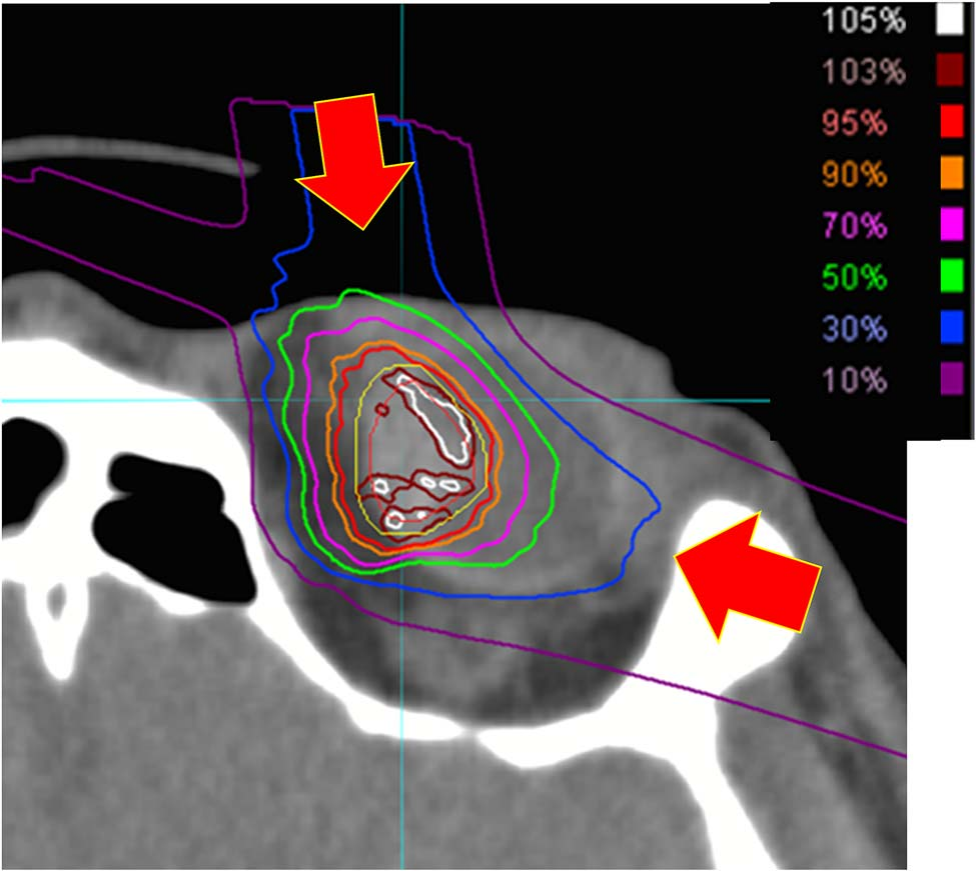
Dose distribution for carbon-ion beam therapy. The arrows indicate treatment direction, one direction per day and two treatments from one direction. A total of four treatments are given.

The gross tumor volume (GTV) was established by findings of ophthalmoscopy, US, CT and MRI, and the clinical target volume (CTV) did not margin with GTV, whereas the planning target volume (PTV) consisted of CTV with a 1.0-mm safety margin for positioning uncertainty. Titanium markers were sutured to the outer surface of the sclera for treatment planning and field localization. Until this study, fixed passive irradiation had been used; however, in this study, a two-port gantry with a scanning beam was used. The typical dose distributions are shown in Fig. 1. Scanning irradiation is a method in which spots with a diameter of 4 mm are placed based on the shape of the tumor, thereby reducing the dose to the proximal side. Furthermore, the use of a gantry enabled irradiation from free angles, which further aided in reducing the dose to normal tissues. The irradiation direction is performed with two-beam irradiation to compensate for the uncertainty of the range of the carbon ion beam. The angles of the two ports are set according to the position of the tumor, and to avoid the uncertainty of passing through the fixation device, the two ports are set to between approximately −10° and 105° for lesions in the left eye. The prescribed dose to PTV was 68 Gy in four fractions over 4 days (relative biological effectiveness weighted dose based on the modified microdosimetric kinetic model) [20, 21].

### Advantages and disadvantages of a rotating gantry with scanning beams

The advantages of this rotating gantry with scanning beams are as follows: (i) scanning irradiation limits the dose to the proximal side, reducing doses to the skin and mucous membranes (Fig. 1); (ii) preparation time is shortened as patient collimators and boluses are not required; (iii) treatment time is reduced due to increased dose rates because patient collimators and boluses are not needed; (iv) the flexible beam angle facilitates dose reduction in the lens and optic nerve papillae. However, a disadvantage of this method is that the sharpness of the penumbra is inferior in scanning irradiation compared to passive irradiation due to the absence of patient collimators.

### DVH analysis

A dose-volume histogram (DVH) analysis of the dose to the iris ciliary body was performed. In accordance with the analysis method of the previous report, the iris ciliary body was defined as the expanding volume of the lens measured 3 mm laterally, adding the volume of the anterior chamber and subtracting the lens volume [12, 19].

## RESULTS

Between April 2018 and July 2019, 21 patients were enrolled in the study and underwent C-ion RT as planned. All 21 patients completed the treatment schedule and the 3 years follow-up periods. The median duration of follow-up was 43 months (range, 35.2–54.6 months). Patient and tumor characteristics are summarized in Table 1.

**Table 1:** Patient and tumor characteristics

		**Total** **21 cases**
Age	Median (range)	62 year (40–85 year)
Sex	male:female	10:11
Follow-up	Median (range)	43 months (36–55 months)
Max. diameter	Median (range)	11.0 mm (6.0 mm–18.5 mm)
Height	Median (range)	5.5 mm (3.5 mm–12.0 mm)
Size category	1:2:3:4	3:11:5:2
Ciliary body invasion	Yes: No	0:21
Optic disk invasion	Yes: No	6:15
Stage (AJCC7th)	I:IIA:IIB:IIIA	3:11:5:2

The acute and late normal tissue responses are presented in Table 2. Grade 2 or higher acute toxicities were not observed. Regarding late normal tissue responses, three of the 21 patients developed grade 2 NVG; however, no other late grade ≥2 toxicities were observed.

**Table 2:** The acute and late normal tissue responses

**Type of adverse event**	**Grade 0–1**	**Grade 2**	**Grade 3–5**
Acute events			
Dermatitis	21	0	0
Eye reaction	21	0	0
Late events			
Dermatitis	21	0	0
Neovascular glaucoma	18	3	0

During the 3-year study period, all 21 patients survived with no local recurrence, and none of these patients underwent enucleation. Three patients showed liver metastasis: one underwent liver tumor resection; one received immune checkpoint inhibitor therapy after C-ion RT for liver metastases; and one received immune checkpoint inhibitor therapy. All the 3-year LC, OS and ER rates were 100%. A visual acuity 20/200 was preserved in 13 of 17 patients (76.5%) with an initial visual acuity 20/200.

As a result of DVH analysis, the median maximum dose to the ciliary body was 17.7 Gy (range 3.8–70.1 Gy), and only one case received a dose of 50 Gy or more.

## DISCUSSION

To the best of our knowledge, this is the first report of C-ion RT for choroidal malignant melanoma using a rotating gantry and scanning irradiation, which was shown to be safe and effective treatment.

**Fig. 2:**
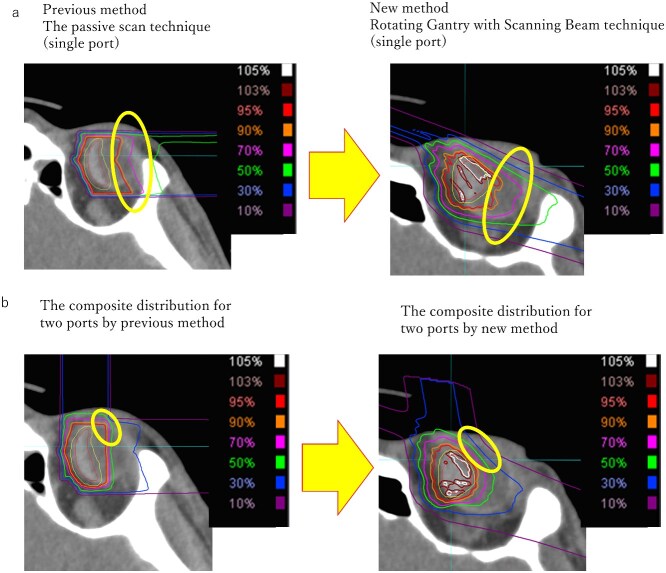
Comparison of the passive scan technique and new method (rotating gantry with scanning beam technique) for dose distribution by single port (a) and 2 port (b) of C-ion RT. The ellipse indicates that the dose in the proximal region is reduced in the new method.

C-ion RT for choroidal malignant melanoma began in April 2001 at NIRS, and several reports have indicated its efficacy and safety [7, 12, 13, 22] (Table 3). In addition, our recent research has also reported that the prescription dose and dose fractionation are not related to the incidence of adverse events or visual acuity preservation [22]. While previous studies used C-ion RT in a passive scan technique with a fixed port, this prospective study used gantry and scanning irradiation. Although the efficacy of the treatment was observed in a small number of cases and the follow-up period was only 43 months, there were no deaths or local recurrences in any of the cases. These results suggested that the therapeutic effect was as good as or better than that of conventional therapy. Although grade 2 skin reactions to acute adverse events was reported in 9% of cases in previous reports [13], there was no incidence of grade 2 dermatitis in this study, indicating that this new treatment method is safe in terms of dose distribution. Scanning C-ion RT may reduce the dose on the proximal side; therefore, the effect on the skin and conjunctiva can be reduced compared to passive C-ion RT, which was the previous irradiation method (Fig. 2). Furthermore, the gantry system may enable safer treatment directions and reduce side effects.

**Table 3:** Previous reports of C-ion RT for Choroidal Malignant Melanoma

**Author**	**Year**	**No. pts**	**Methods of C-ion RT**	**Dose/fraction**	**Median follow-up duration (month)**	**Incidence of grade 2 or more NVG**	**Local control rate at 3 year**
Tsuji *et al.* [13]	2007	59	Conventional passive irradiation methods	60–85 Gy/5fr.	26.1	52.8%	97.4%
Hirasawa *et al.* [19]	2007	55	Conventional passive irradiation methods	60–85 Gy/5fr.	35.0	42.6%	NA
Toyama *et al.* [12]	2013	116	Conventional passive irradiation methods	60–85 Gy/5fr.	55.2	41.6% (1 port) 13.9% (2 ports)	95.7%
Aoki *et al.* [22]	2025	250	Conventional passive irradiation methods and rotating gantry with scanning beams	60–85 Gy/5fr. 64–68 Gy/4fr.	72.5	21.6%	94.4% (5-year)
Current Study		21	Rotating gantry with scanning beams	68 Gy/4fr.	43.0	18.6%	100%

Regarding late adverse events, our previous study reported that the incidence of grade 2 NVG at 3 years was 41.6% and 13.9% for 1- and 2-portal irradiation, respectively, and that irradiation of the iris and ciliary body was associated with the development of NVG. In this novel method, it is possible to reduce the proximity dose of GTV by scanning irradiation (Fig. 2) because the treatment angle can be freely selected using the rotating gantry, and the dose to the iris-ciliary body can be reduced. In our previous report, we reported that the median V50 (the volume irradiated ≥50 Gy) was 0.127 ml (range, 0–0.356 ml) in an analysis of 55 cases of previous treatment methods, and V50 was a factor related to the development of NVG, in this study, only one of the 21 cases received a dose of 50 Gy or more, and the other cases received a dose of ˂50 Gy. On the other hand, the 3-year incidence of NVG was 18.6% in the previous study, similar to previous reports on 2-portal irradiation. This may be due to the small number of cases and the different localization of the tumors. Since the factor that most influences iris ciliary body dose is tumor localization, it is necessary to accumulate more cases with this new irradiation method, compare it with conventional irradiation methods and analyze more optimal irradiation methods.

The 3-year ERR in this trial was 100% because local control had been achieved in all patients, and no grade 3 or higher NVG had appeared at 3 years, which is comparable with or better than the 3-year ERR of 96.1% reported in a previous study on C-ion RT [12]. This is attributed to the reduction in the ciliary body dose and corneal and conjunctival doses using the new irradiation method. Conventional passive C-ion RT has 5-year local control and ERR of 93% and 93%, respectively, which are comparable to those of proton radiotherapy and stereotactic radiotherapy [9, 19, 20].

In terms of vision preservation, a visual acuity 20/200 was preserved in 13 of 17 patients (76.5%) with an initial visual acuity 20/200. In our recent analysis of 251 cases, the thickness of the tumor and the distance from the macular to the optic disc were found to be significant factors in preserving visual acuity [22]. The number of cases in this study was small, and it is possible that there were relatively many favorable cases, so we would like to increase the number of cases and conduct further analysis in the future.

The limitation of this study is that it is a report of a small number of cases and the follow-up period is relatively short. Although only 21 patients were treated in this study and the follow-up period was only 43 months, local control and ERR were achieved in all patients, suggesting that the use of a rotating gantry with scanning beam of C-ion RT may achieve equivalent local control and ERR. Further evidence can be obtained by increasing the number of cases and extending the follow-up period. In addition, to clarify the effectiveness of this new treatment method, it is necessary to compare the dose distribution with the conventional treatment method, and we plan to conduct a dose comparison study using the same dose calculation algorithm in the future.

The results of this prospective study confirmed the acute safety and suggested comparable effectiveness and long-term toxicity to previous reports using passive scattering particle (hadron) therapy. Further validation by increasing the number of cases and extending the observation period is necessary to evaluate the efficacy and safety of C-ion RT in comparison with proton therapy, which is the worldwide standard.

## Presentation at a conference

None.

## Clinical trial registration number

http://www.umin.ac.jp/ctr/index-j.htm; identification number: UMIN000031715.

## Data Availability

The data that support the findings of this study are not openly available due to reasons of sensitivity and are available from the corresponding author upon reasonable request. Data are located in controlled access data storage at QST hospital.
